# Cognitive Demands and Emotional Responses in L2 Writing: How Task Complexity and Task-Specific Emotions Shape Learners’ Performance

**DOI:** 10.3390/bs16040580

**Published:** 2026-04-12

**Authors:** Yue Wang, Yunmei Sun, Wen Ke

**Affiliations:** School of Foreign Languages, Huazhong University of Science and Technology, Wuhan 430074, China; d202582140@hust.edu.cn (Y.W.); m202475488@hust.edu.cn (W.K.)

**Keywords:** task complexity, task-specific emotion, L2 writing, cognitive hypothesis, control-value theory

## Abstract

This study examined the complex relationships among task complexity, task-specific emotions, and learners’ L2 writing performance. Sixty-one Chinese EFL learners were divided into two groups and completed two writing tasks in a counterbalanced order. They reported their perceptions about task complexity and their emotional experience during the task after completing each task. The results revealed that (1) task complexity exerted a significant negative effect on learners’ writing performance in terms of content, but no significant effects on language use, organization and communicative achievement, or overall writing performance; (2) task complexity did not significantly affect learners’ task enjoyment, task anxiety or task boredom; (3) task-specific emotions did not predict learners’ overall writing performance in the simple task, while task anxiety exerted a significant negative effect on learners’ performance in the complex task. These results are discussed with reference to the Cognition Hypothesis and the Control-Value Theory, particularly the intensified role of affect under increased cognitive demands and the negative impact of task anxiety on writing performance, offering pedagogical implications for L2 writing.

## 1. Introduction

Second language (L2) writing is a complex cognitive process shaped by the interplay between task environment and individual differences (Flower & Hayes, 2004; C. Li et al., 2024). As one of the key elements of the task environment, task complexity refers to the cognitive demands imposed by a task’s structure upon learners’ attentional, memory, and reasoning resources (Robinson, 2001a). In recent years, the effect of task complexity on L2 writing performance has become a subject of considerable interest in the field of second language acquisition (SLA). However, existing findings remain inconclusive, particularly regarding how task complexity influences different dimensions of writing performance (Shen et al., 2025). Moreover, prior work has primarily examined L2 writing performance in terms of linguistic features such as complexity, accuracy, and fluency (CAF), while often overlooking the communicative goals of writing tasks (Xu et al., 2023). These limitations indicated the need for a multidimensional assessment of task complexity effects on L2 writing performance (Rahimi, 2019; Rahimi & Zhang, 2018). Although pioneering studies have explored the impact of task complexity on multidimensional outcomes, including CAF, content, and organization (Rahimi, 2019; Rahimi & Zhang, 2018), empirical evidence remains relatively limited. There is therefore a need to extend this line of inquiry to broader educational contexts and populations and to incorporate more indicators of writing performance, such as communicative achievement.

In addition to the cognitive demands of tasks, the roles of learners’ emotions (e.g., enjoyment, anxiety, and boredom) in L2 writing have attracted increased scholarly attention (Abdi Tabari et al., 2024, 2025; Aubrey, 2025; C. Li, 2024; C. Li et al., 2024; C. Li & Wei, 2023; Pawlak et al., 2025; Rahimi & Zhang, 2019; Wu & Halim, 2024; Yang et al., 2025). Traditionally, emotions are conceptualized as stable and trait-like constructs in SLA (Dewaele & Alfawzan, 2018; Dewaele & MacIntyre, 2014). More recent work has begun to differentiate short-term emotional responses that emerge during tasks (i.e., task-specific emotions) from trait emotions (Aubrey, 2025; C. Li et al., 2024; C. Li & Dewaele, 2024; Rahimi & Zhang, 2019). Highlighting task-specific emotions allows researchers to capture learners’ moment-to-moment affective fluctuations that more directly shape their engagement with writing processes (Rahimi & Zhang, 2019). Despite growing interest in this area, empirical evidence remains limited regarding how task-specific emotions interact with task complexity to influence multidimensional writing performance (C. Li et al., 2024).

In light of these gaps, the present study builds upon previous research (C. Li et al., 2024; Rahimi, 2019; Rahimi & Zhang, 2018, 2019) by broadening both the affective scope and the assessment framework. Specifically, it examines the interplay among task complexity; a wider range of task-specific emotions including enjoyment, anxiety, and boredom; and Chinese university students’ L2 writing performance across multiple dimensions, namely language use, content, organization, and communicative achievement. By adopting a task–individual interaction perspective and a multidimensional approach to writing assessment (Flower & Hayes, 2004; C. Li et al., 2024; Xu et al., 2023), this study seeks to advance understanding of how cognitive and affective factors jointly shape L2 writing and to provide pedagogical insights for task design and writing instruction.

## 2. Literature Review

### 2.1. Task Complexity and L2 Writing

The two most prevalent theories regarding how task complexity affects language production are Skehan’s (1998) Trade-off Hypothesis and Robinson’s (2001a, 2001b, 2010) Cognition Hypothesis. The fundamental assumption of the Trade-off Hypothesis (Skehan, 1998) is that learners’ attentional resources available during tasks are limited. Therefore, when the cognitive demands of tasks become more complex, learners are unable to allocate sufficient attention to complexity, accuracy, and fluency equally. Skehan (1998) further notes that as task complexity increases, learners tend to pay more attention to the comprehensibility of their linguistic output, prioritizing fluency over complexity or accuracy.

By contrast, Robinson (2001a, 2001b, 2010) claims that learners possess multiple cognitive resource pools for different dimensions of their linguistic output. Robinson further argues that task complexity can be divided into two distinct dimensions, i.e., the resource-directing dimension and the resource-dispersing dimension. Increasing task complexity along the resource-directing dimension, such as increasing reasoning demands, promotes learners’ attention to form-meaning mapping and leads to the improvement of accuracy and complexity of their linguistic output (C. Li et al., 2024). Increasing task complexity along the resource-dispersing dimension, such as decreasing planning time, imposes performative and procedural demands on learners and enhances the automatic retrieval and use of existing linguistic knowledge.

Although both the Trade-off Hypothesis and the Cognition Hypothesis originally targeted L2 oral production (Robinson, 2001a, 2001b, 2010; Skehan, 1998), they were soon extended to studies of L2 writing (Cho, 2018; Golparvar & Rashidi, 2021; Lee, 2020; C. Li et al., 2024; Rahimi, 2019; Rahimi & Zhang, 2018, 2019; Teng & Zhan, 2023; Wu & Halim, 2024; Xu et al., 2023; Yang et al., 2025). However, given the disparities in research design, subject groups, and writing metrics, existing research findings are inconsistent (Shen et al., 2025). For example, Xu et al. (2023) found that increasing task complexity did not significantly affect syntactic complexity, accuracy, or fluency, but it reduced lexical complexity. In contrast, Golparvar and Rashidi (2021) reported that task complexity significantly improved several measures of syntactic complexity, increased lexical diversity, while negatively affecting accuracy.

Another limitation of existing research is its predominant reliance on CAF to assess learners’ writing performance (e.g., Adams et al., 2015; Lee, 2020; Liu et al., 2023; Révész et al., 2017; Tabari et al., 2023; Wu & Halim, 2024). However, tasks are inherently goal-directed and content-centered activities (Richards & Schmidt, 2013). Accordingly, task-based language assessment should primarily attend to the “communicative performance” (Ellis, 2003, p. 279) of learners’ language use within specific contexts, i.e., whether the intended task goals have been successfully achieved (Shehadeh, 2012, 2018). The problem of the sole reliance on CAF measures is that learners may produce linguistically sophisticated texts while still failing to accomplish the task goals (e.g., producing irrelevant content or using the inappropriate style for a certain genre) (Xu et al., 2023). Another concern is that increased cognitive demands may reduce linguistic performance while simultaneously enhancing non-linguistic aspects of writing. For instance, Rahimi (2019) and Rahimi and Zhang (2018) systematically investigated the effects of increasing reasoning demands and the number of elements on L2 argumentative writing. Their findings revealed that while increased cognitive task complexity exerted a negative effect on accuracy and fluency, it simultaneously enhanced syntactic and lexical complexity, as well as higher-order dimensions such as content, organization, and overall text quality. These findings highlight the necessity of looking beyond linguistic metrics and a multidimensional approach to task-based language assessment.

### 2.2. Task-Specific Emotions and L2 Writing

The Control-Value Theory (Pekrun, 2006; Pekrun & Perry, 2014) offers an integrated theoretical framework for understanding the antecedents and outcomes of emotions related to academic achievements in educational settings. The core assumption of the Control-Value Theory is that emotions are largely determined by learners’ control appraisals (the perceived controllability of learning activities and outcomes) and value appraisals (the perceived importance of learning activities), which in turn, affect their cognition, motivation, engagement and learning outcomes (C. Li et al., 2024; Pawlak et al., 2025; Pekrun, 2006; Pekrun & Perry, 2014).

Building on the Control-Value Theory, C. Li (2024) further distinguishes three levels of emotions within the L2 learning process according to their situation specificity, including the general domain-level (i.e., foreign language learning anxiety), the skill-specific level (writing anxiety), and the task-specific level (task anxiety). While general domain emotions reflect a learner’s long-term emotional tendencies, task-specific emotions offer immediate, context-dependent insights into how students engage with specific assignments (C. Li, 2024; Pawlak et al., 2025). However, research in SLA has long focused on trait-like emotions at the general-domain level (Dewaele & Alfawzan, 2018; Dewaele & MacIntyre, 2014; C. Li & Wei, 2023) or skill-specific level (Güvendir & Uzun, 2023; Papi, 2022), leaving the dynamic nature of task-specific emotions relatively underexplored (C. Li, 2024; C. Li et al., 2024). Addressing this gap is critical. Although stable trait-like emotions and task-specific emotions can co-occur and correlate with each other in a task, empirical evidence suggests that only the latter directly predict L2 writing performance (C. Li et al., 2024). Therefore, to accurately capture the affective drivers of task-based writing outcomes, it becomes particularly important to investigate emotions at the task-specific level.

The present study focuses specifically on task enjoyment, task anxiety, and task boredom, as they are the most frequently experienced and influential emotions in L2 writing contexts (Pawlak et al., 2025; Yang et al., 2025). According to the three-dimensional taxonomy of the Control-Value Theory (Pekrun, 2006; Pekrun & Perry, 2014), these emotions exhibit distinct characteristics and roles in L2 writing tasks. Task enjoyment is a positive, activating, and activity-oriented emotion. It is believed to stimulate learners’ interest and motivation, enabling them to effectively cope with task demands and thereby improving L2 writing performance (C. Li, 2024; C. Li et al., 2024; Papi, 2022). Conversely, task anxiety is a negative, activating, and outcome-oriented emotion that occurs when learners perceive a task as excessively difficult or experience diminished control (Pawlak et al., 2025; Rahimi & Zhang, 2019). Task anxiety may divert learners’ attention, undermine motivation and behavioral engagement, and hinder writing performance (C. Li et al., 2024; Pawlak et al., 2025; Yang et al., 2025). Finally, task boredom is a negative, activity-oriented emotion characterized by a low level of activation. It is often triggered by monotonous tasks or a disconnection from learner interests, leading to reduced engagement and superficial information processing (Pekrun & Perry, 2014).

In summary, task-specific emotions are critical to learners’ cognition, motivation, behavioral engagement, and the final writing performance. Given the paucity of related empirical studies, it is essential to further examine not only their direct effects but also how they interact with the task environment to shape learners’ performance.

### 2.3. Task Complexity, Task-Specific Emotion, and L2 Writing

The Cognition Hypothesis (Robinson, 2001a, 2001b, 2010) and the Control-Value Theory (Pekrun, 2006; Pekrun & Perry, 2014) build the theoretical foundation for bridging task complexity and task-specific emotions in L2 writing. According to the Control-Value Theory, task demands (i.e., task complexity) serve as the distal antecedents to achievement emotions by shaping learners’ perceived task control and task value (Pekrun, 2006). On the one hand, a complex task (with more elements and higher reasoning demands) may undermine a learner’s sense of control, leading to high levels of negative activating emotions like anxiety and reducing task performance. On the other hand, task complexity influences value appraisals based on the match between the cognitive demands and learners’ capabilities (Yang et al., 2025). If tasks are perceived as presenting an optimal challenge that aligns with learners’ capabilities, the incentive value and perceived controllability of the activity will be enhanced, potentially resulting in the positive, activating emotion of enjoyment and improved task performance (Pekrun, 2006). The Cognition Hypothesis (Robinson, 2001a, 2001b, 2010) also posits that learner affect interacts with task complexity to promote or mitigate task performance. It further assumes that learner affect would exert a stronger effect on task performance with the increase in cognitive demands (Robinson, 2010). Consequently, the impact of task-specific emotions on L2 writing is theoretically expected to be context-dependent, becoming more evident under complex conditions.

Despite the theoretical claims on the interplay among task complexity, task-specific emotions, and task performance, empirical research remains scarce (C. Li et al., 2024). While Rahimi and Zhang (2019) pioneered the investigation of the links between task complexity and anxiety among upper-intermediate EFL learners in private language schools in central Iran, C. Li et al. (2024) expanded this affective scope by pioneering an exploration of task-specific enjoyment and L2 writing performance among young learners in a rural Chinese junior secondary school. However, empirical evidence involving more diverse populations (e.g., university students in central China) and examining a more comprehensive set of task-specific emotions (e.g., enjoyment, anxiety, and boredom) is still lacking. Therefore, building on the aforementioned theoretical framework and empirical studies, the present study will address the following questions:

1. What are the effects of task complexity on learners’ L2 writing performance (in terms of content, organization, language, and communicative ability)?
2. What are the effects of task complexity on learners’ task enjoyment, task anxiety, and task boredom?
3. How do task-specific emotions predict learners’ L2 writing performance in simple and complex tasks?

## 3. Methodology

### 3.1. Research Design

**Figure 1:**
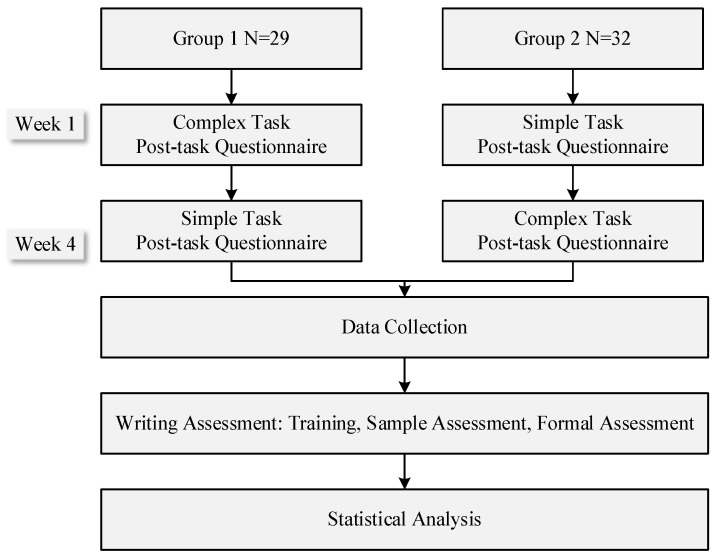
Research Procedure.

The research design and experimental procedure are shown in Figure 1. This study used a counterbalanced design to prevent the order effect (Jung, 2018), which is statistically validated through a series of mixed ANOVA analysis for both writing performance and task-specific emotions, confirming the absence of any significant condition-order interaction effects (overall writing performance: *F* (1,59) = 0.575, *p* = 0.451; task-specific enjoyment: *F* (1,59) = 0.021, *p* = 0.886; task-specific anxiety: *F* (1,59) = 3.424, *p* = 0.069; task-specific boredom: *F* (1,59) = 1.088, *p* = 0.301). Participants in each group completed the writing tasks in either a simple-to-complex or complex-to-simple sequence. Following each writing task, participants completed a post-task questionnaire assessing their perceptions of task difficulty, cognitive effort, and task-specific emotions. The two tasks were scheduled three weeks apart, consistent with prior writing literature that aligns pre- and post-tests with regular course weeks (Wilby, 2020), and this arrangement can help reduce the potential cognitive fatigue associated with massed practice for learners (Heidari, 2025). Writing samples were initially collected on paper and then transcribed into digital documents, while questionnaire data were gathered via the online platform wenjuanxing. Following data collection and preliminary screening, trained raters assessed the writing samples. Finally, the post-task questionnaire data and writing performance scores were imported into SPSS Version 29 (IBM Corp., 2022) for statistical analysis.

### 3.2. Participants

Sixty-two participants were initially recruited from a university in central China. Following data screening (see Section 3.4 for details), one participant was excluded, resulting in a final sample of 61 participants. Their ages ranged from 17 to 19 years (M = 18.23, SD = 0.46), including 51 males and 10 females. Among them, 29 majored in Computer Science and 32 in Electrical Engineering. All participants were native speakers of Chinese with more than ten years of English learning experience. Their average score on the National College Entrance Examination English test was 137 out of 150, indicating an upper-intermediate level of English proficiency.

### 3.3. Research Instruments

#### 3.3.1. Writing Task

The argumentative writing tasks of this study (see Appendix A) were from Xu et al. (2023). In the simple task, participants were required to select two roommates from four candidates, each described by four attributes (e.g., personality, daily routines, interests, and learning styles). In the complex task, participants were required to select four optimal roommates from six candidates, each described by six attributes (e.g., personality, daily routines, interests, learning styles, subject preference, and sanitary habits). Both tasks required participants to write an approximately 200-word argumentative essay within 40 min and justify their choices with supporting ideas.

#### 3.3.2. Rating Criteria and Procedure

This study adopted the writing assessment criteria of the Cambridge B2 First exam to evaluate learners’ task performance. The assessment consisted of four subscales, including language, content, organization, and communicative achievement. Each subscale ranges from 0 to 5, and the overall score for task performance ranges from 0 to 20.

The rating procedure was conducted in three stages involving four trained raters, all of whom are teachers of English in a university who have at least five years of experience rating a nationwide standardized English test in China. First, the first author provided a comprehensive briefing to all raters regarding the specific task requirements and the scoring criteria. Second, the raters assessed three samples independently and then compared their results in a group discussion to further refine the rating criteria (see Appendix A). Finally, in the formal scoring stage, all handwritten scripts were transcribed into anonymized digital documents. Each script was independently rated by two raters, and the final score for each dimension was derived by averaging the two raters’ scores. Because scripts were anonymized and randomly distributed across the rating pool, raters were blind to participant identities and unlikely to encounter the two scripts from the same participant in close succession, thereby minimizing potential memory effects.

The 122 scripts analyzed in the present study were drawn from a larger research project in which each of the four raters evaluated approximately 100 scripts. Owing to this predefined workload allocation, the scripts included here fell into two distinct rating assignments: scripts 1–19 were evaluated by one rater pair, whereas scripts 20–122 were evaluated by another pair. The resulting data structure therefore constituted an incomplete two-way design. Inter-rater reliability was estimated using an intraclass correlation coefficient of interrater consistency, ICC (Q, $\hat{K}$), following the framework for incomplete two-way design proposed by Ten Hove et al. (2024). Estimates obtained via maximum likelihood in R version 4.5.2 (R Core Team, 2025) indicated strong Inter-rater reliability across subscales: content ICC = 0.895 (95% CI [0.849, 0.928]), organization ICC = 0.892 (95% CI [0.845, 0.926]), language ICC = 0.818 (95% CI [0.738, 0.875]), and communicative achievement ICC = 0.817 (95% CI [0.736, 0.874]).

#### 3.3.3. Task Complexity Manipulation and Validation

It is well documented in the literature that tasks involving more elements and higher reasoning demands impose a greater cognitive load on learners (C. Li et al., 2024; Rahimi, 2019; Rahimi & Zhang, 2018, 2019). Accordingly, the present study manipulated task complexity through these two prevailing resource-directing variables, i.e., the number of elements and the reasoning demands.

Informed by prior literature (Rahimi, 2019; Rahimi & Zhang, 2018, 2019), the manipulation of task complexity is then validated through both expert judgment and participant perceptions. First, two experts in SLA evaluated the task design and agreed that the complex task imposed higher difficulty and cognitive effort. Second, participants completed a questionnaire assessing their perceived task difficulty and mental effort after each task. The scale (Révész et al., 2017) included two nine-point Likert items: “This task was very difficult” and “This task required a great deal of cognitive effort.” For task difficulty, paired-samples t-tests indicated that the complex task (M = 4.53, SD = 1.91) was perceived as significantly more difficult than the simple task (M = 3.81, SD = 1.48), with a modest effect size (*t* (60) = −2.332, *p* = 0.023, 95% CI [−1.340, −0.103], *d* = 0.30). For mental effort, although the difference did not reach statistical significance (*t* (60) = −1.754, *p* = 0.084, 95% CI [−1.368, 0.090], *d* = 0.22), the mean rating was consistently higher in the complex task (M = 5.00, SD = 1.90) than in the simple one (M = 4.36, SD = 1.77).

#### 3.3.4. Task-Specific Emotion Scale

A subset of 14 items was adopted from the task-specific emotion scales developed by C. Li et al. (2024) and C. Li (2024). The selected items comprised three constructs: task enjoyment (five items), task anxiety (five items), and task boredom (four items), each measured on a nine-point Likert scale (see Appendix B). Because the original scale was developed for secondary school students, an exploratory factor analysis (EFA) was conducted to establish its structural validity within a university sample. To satisfy the assumption of independent observations in this within-subject design, the EFA was conducted using responses from the simple-task condition, which serves as the baseline prior to increasing task complexity. The Kaiser-Meyer-Olkin Measure was 0.780, and Bartlett’s test of sphericity was significant (*p* < 0.001), indicating suitability for factor analysis. Principal Axis Factoring with Promax rotation was employed, and factors were retained based on eigenvalues greater than 1 and the scree plot. The analysis yielded a clear three-factor solution corresponding to enjoyment, anxiety, and boredom, explaining 71.82% of the total variance. All items loaded on their intended factors, with a pattern matrix ranging from 0.457 to 0.974, supporting the construct validity in the present study.

Based on this established factor structure, internal consistency reliability was examined for both task conditions. Cronbach’s alpha coefficients indicated high reliability for enjoyment (simple α = 0.872; complex α = 0.796), anxiety (simple α = 0.943; complex α = 0.932), and boredom (simple α = 0.861; complex α = 0.827), demonstrating stable internal consistency across task conditions.

### 3.4. Data Analysis

After the collection of questionnaire data and writing samples, data screening was conducted. One participant failed to submit a complete writing sample for the complex tasks. To ensure matched observations across both task conditions, this participant’s data was entirely excluded, resulting in a final dataset of 122 valid questionnaires and 122 writing samples from 61 participants for the writing assessment and statistical analysis.

Before formal analysis, normality tests were conducted for all core variables by examining skewness and kurtosis (Kline, 2011). The results indicated that skewness values were within ±3 and kurtosis values were within ±10 (Table 1), suggesting that the data were approximately normally distributed and suitable for parametric statistical analyses. For Research Questions 1 and 2, paired-samples *t*-tests were conducted to examine differences between simple and complex tasks regarding learners’ task performance and task-specific emotions. For Research Question 3, Pearson correlation analyses and regression analyses were conducted separately under simple and complex task conditions to investigate the predictive effects of task-specific emotions on overall writing performance. All the aforementioned data analyses were performed using SPSS Version 29 (IBM Corp., 2022).

## 4. Results

### 4.1. Effects of Task Complexity on Learners’ L2 Writing Performance

The paired-samples *t*-test showed that task complexity only exerted a significant effect on the content of learners’ writing performance (see Table 2). Specifically, participants obtained higher content scores in the simple task than in the complex task (*M* diff = 0.19, 95% CI [0.00, 0.38], *t* (60) = 2.014, *p* = 0.049, *d* = 0.258). To address the potential inflation of Type I error from testing multiple subscales, a Bonferroni correction was applied (α = 0.01). Under this stricter threshold, the difference in content performance did not remain statistically significant. Therefore, this specific reduction should be interpreted with caution as a tentative trend rather than a definitive effect.

In contrast, no significant differences were observed for the overall writing score (*M* diff = 0.33, 95% CI [−0.15, 0.80], *t* (60) = 1.384, *p* = 0.172, *d* = 0.177), nor for the other subscales: organization (*M* diff = 0.15, 95% CI [−0.08, 0.37], *t* (60) = 1.321, *p* = 0.192, *d* = 0.169), language (*M* diff = −0.01, 95% CI [−0.14, 0.12], *t* (60) = −0.127, *p* = 0.899, *d* = −0.016), and communicative achievement (*M* diff = 0.00, 95% CI [−0.14, 0.14], *t* (60) = 0.000, *p* = 1.000, *d* = 0.000). These results indicate that learners’ performance in these dimensions remains generally stable across different task conditions.

### 4.2. Effects of Task Complexity on Learners’ Task-Specific Emotions

The paired-samples t-test results revealed no significant differences for the three task-specific emotions in simple and complex tasks (see Table 2). Although descriptive trends pointed to slightly lower enjoyment (*M* diff = 0.04, 95% CI [−0.44, 0.52], *t* (60) = 0.164, *p* = 0.870, *d* = 0.021) and boredom (*M* diff = 0.06, 95% CI [−0.50, 0.61], *t* (60) = 0.206, *p* = 0.837, *d* = 0.026), along with higher anxiety (*M* diff = −0.05, 95% CI [−0.79, 0.69], *t* (60) = −0.133, *p* = 0.894, *d* = −0.017) in the complex task, these differences failed to reach statistical significance. The effect sizes were also negligible, suggesting that task complexity had minimal impact on learners’ task-specific emotions.

### 4.3. Effects of Task-Specific Emotions on Learners’ L2 Writing Performance in Simple and Complex Tasks

As shown in Table 3, Pearson correlation analyses indicated that none of the task-specific emotions were significantly correlated with the overall L2 writing performance in the simple task (*p* > 0.05). Following the principle of parsimony, the regression analysis was not pursued for this condition due to the lack of significant initial bivariate relationships.

In the complex task, however, task anxiety showed a significant negative correlation with writing performance (*r* = −0.284, *p* = 0.027), whereas no significant correlations were found for task enjoyment (*p* = 0.783) or task boredom (*p* = 0.546). To further examine the predictive effects of emotions on writing performance in this condition, a stepwise multiple regression analysis was conducted. All three emotions (enjoyment, anxiety, and boredom) were initially entered into the model as potential predictors. The results (see Table 4) indicated that the final regression model was significant (*F* (1, 59) = 5.162, *p* = 0.027), with task anxiety alone accounting for 8.0% of the variance in overall writing performance (*R*^2^ = 0.080). Task enjoyment (beta = −0.055, *p* = 0.678) and task boredom (beta = 0.088, *p* = 0.487) were excluded from the final model as they did not make statistically significant contributions. Furthermore, regression assumption checks indicated no issues with multicollinearity, with Tolerance values ranging from 0.908 to 1.000 and Variance Inflation Factor (VIF) values well below the standard threshold of 5 (ranging from 1.000 to 1.101) across all tested predictors.

## 5. Discussion

### 5.1. Effects of Task Complexity on Learners’ L2 Writing Performance

In response to Research Question 1, the present study found that task complexity had a significant negative effect on learners’ L2 writing performance in terms of content. This can be viewed as the result of the intensified competition for cognitive resources among different stages of the writing process. Writing is a cognitive activity involving processes such as text interpretation, reflection, and text production, all of which draw on cognitive resources including short-term working memory and long-term memory (Hayes, 2000). When the elements and reasoning demands of a task increase, learners have to allocate more working memory resources to the process of text interpretation (e.g., reading the task material) to cope with the higher cognitive demands. Consequently, the working memory resources remaining for reflection and text production become constrained. Because semantic content generation during the text production stage also depends on working memory resources (Hayes, 2000), diverting these resources to other writing components may reduce the adequacy and depth of learners’ content performance.

In contrast, task complexity did not have a significant effect on the language, organization, communicative achievement, or overall writing performance. This indicates a sufficient alignment between learners’ competence and the requirements of the rating criteria. According to the Cambridge English B2 First writing rubric, indicators for language, organization, and communicative achievement essentially assess learners’ use of linguistic, discourse, and genre knowledge stored in long-term memory (Flower & Hayes, 2004; Hayes, 2000). For upper-intermediate L2 learners, the relevant writing knowledge and skills can become proceduralized (Lightbown & Spada, 2013) through sustained practice and stored in long-term memory. This allows for automated processing during writing, minimizing reliance on working memory. Consequently, learners’ performance in these areas tends to remain relatively stable even when task complexity increases.

The present study found that while task complexity had no significant effect on learners’ language performance in L2 writing, it significantly reduced learners’ content performance, echoing the findings of Xu et al. (2023). These results suggest that learners cannot distribute cognitive resources equally across multiple dimensions in the complex task (C. Li et al., 2024), which generally aligns with the central claim of the Trade-off Hypothesis (Skehan, 1998).

### 5.2. Effects of Task Complexity on Learners’ Task-Specific Emotions

In response to Research Question 2, the present study found that task complexity has no significant effects on learners’ task enjoyment, task anxiety, and task boredom. Several possible explanations may account for this result.

First, the influence of task complexity may be associated with learners’ perceived cognitive load (J. Li & Wang, 2017). Although participants perceived the complex task as more demanding, they reported comparable levels of mental effort across the two tasks. Given their upper-intermediate level of English proficiency, the cognitive load induced by the complex task may not have exceeded the threshold required to produce statistically observable emotional changes (Xing et al., 2024). As a result, learners’ task-specific emotions remained stable across the two task conditions.

Second, interactions between other task features (pre-post design) and learners’ control-value appraisal may also reduce the influence of task complexity. The two tasks shared the same topic (roommate selection) and a similar structure, which enables learners with a higher degree of familiarity and a sense of control during the second task session (Abdi Tabari et al., 2025). The familiarity and perceived task control typically promote enjoyment and reduce anxiety (Abdi Tabari et al., 2024; Aubrey et al., 2022; Lambert & Zhang, 2019), thereby partially neutralizing the expected emotional differences between simple and complex tasks.

In addition, the roommate selection tasks used in this study are characterized by a topic highly relevant to real-life contexts (Aubrey et al., 2022; Lambert & Zhang, 2019) and offer learners choices (Nakamura et al., 2021). These task features are known to enhance learners’ perceived task value (Aubrey, 2025), elicit learners’ task enjoyment (Aubrey, 2017), and reduce task boredom (Pekrun, 2006) in both simple and complex tasks, which may further contribute to the nonsignificant emotional differences observed across the two task conditions.

The findings of this study align with those of Xing et al. (2024), who also observed that task complexity has no significant impact on learners’ task-specific emotions. However, C. Li et al. (2024) report a small but significant positive effect of task complexity on task enjoyment. The discrepancy could be attributed to differences in participants’ developmental stages and their corresponding emotional regulation abilities. Neuroscience research indicates that emotional regulation ability increases with age and cognitive maturation (Lewis et al., 2010; McRae, 2009). Unlike secondary school students in C. Li et al. (2024), the university students in both Xing et al. (2024) and the present study possess more advanced regulatory mechanisms, thus offsetting the potential influence of increased task complexity on emotions.

### 5.3. Effects of Task-Specific Emotions on Learners’ L2 Writing Performance in Simple and Complex Tasks

In response to Research Question 3, the results showed that task-specific emotions did not significantly affect learners’ L2 writing performance in the simple task, which is consistent with prior findings (C. Li et al., 2024; Rahimi & Zhang, 2019; Yang et al., 2025). This could be attributed to learners’ use of self-regulating writing strategies (Teng & Zhan, 2023). Teng and Zhan (2023) argue that learners demonstrate stronger self-regulation in simple writing tasks, allowing them to better cope with the cognitive demands of the task (Yang et al., 2025). As a result, the impact of task complexity is diminished by learners’ proactive cognitive regulation (Yang et al., 2025).

In contrast, task anxiety exerted a significant negative impact on writing performance in the complex task, aligning with the findings of C. Li et al. (2024) and Yang et al. (2025). This result lends further support to the Control-Value Theory (Pekrun, 2006; Pekrun & Perry, 2014), which posits that learners’ emotional experiences and subsequent task performance are shaped by their appraisals of task control and task value. As task complexity increases, learners may experience heightened uncertainty about the success of writing, thereby reducing the perceived control and triggering task anxiety. As a highly activating and persistent negative emotion (C. Li, 2024; Pawlak et al., 2025; Pekrun & Perry, 2014), task anxiety may undermine learners’ writing performance by eliciting task-irrelevant thoughts (e.g., worry about the potential failure), weakening motivation, and diminishing both cognitive and behavioral engagement (C. Li, 2024; Yang et al., 2025). Meanwhile, task enjoyment did not yield the expected improvement in writing performance, which may be explained by the offsetting influence of anxiety’s negative effects. Task boredom also failed to exert a significant effect, suggesting that learners generally perceived the tasks as valuable and remained sufficiently engaged throughout the task performance (Pekrun, 2006).

Collectively, these findings show that the impact of task-specific emotion on writing performance is sensitive to task complexity (Rahimi & Zhang, 2019). While task-specific emotions play a limited role in the simple task, the negative influence of task anxiety becomes more pronounced as task demands increase. This pattern aligns with the Cognition Hypothesis (Robinson, 2001a, 2001b, 2010), which argues that learner affect would demonstrate a stronger effect in more demanding tasks. This suggests that in more cognitively demanding writing tasks, instructors may need to provide additional emotional support to help learners manage heightened anxiety and maintain effective engagement.

## 6. Conclusions, Limitations, and Future Directions

Grounded in the Cognition Hypothesis (Robinson, 2001a, 2001b, 2010) and the Control-Value Theory (Pekrun, 2006; Pekrun & Perry, 2014), this study examined how task complexity and task-specific emotions jointly shape learners’ L2 writing performance. The findings showed that increased task complexity exerted a significant negative effect on learners’ content performance, while its effects on language, organization, and communicative achievement were not significant. Moreover, although task complexity did not directly influence learners’ emotional experience, it affected how emotions related to writing performance. Specifically, the effect of task anxiety on writing performance became significant as the cognitive demands increased.

Theoretically, these findings provide empirical support for the Cognition Hypothesis (Robinson, 2001a, 2001b, 2010) and the Control-Value Theory (Pekrun, 2006; Pekrun & Perry, 2014), and contribute to a more comprehensive understanding of how learners interact with task environments to influence L2 writing outcomes. Pedagogically, the findings offer tentative evidence that aligning task demands with learners’ capacity may support learners’ engagement and emotional experience. When designing and implementing a task, teachers could consider learners’ individual differences to make tasks appropriately challenging, so that learners might feel more engaged and less anxious or bored. Additionally, selecting task topics that resonate with learners’ interests may enhance perceived task value and foster positive emotions such as enjoyment, which could in turn contribute to better engagement and improved writing outcomes.

The present study also has limitations. First, the reliance on retrospective self-reports introduces subjectivity and may not fully capture the dynamic nature of emotions, suggesting a need for future process-oriented measures to trace real-time fluctuations. Second, while the present study validated learners’ perceived difficulty of the two tasks, the cognitive load induced by both tasks may be similarly manageable for these upper-intermediate learners. Therefore, we recommend that future studies establish and validate varying degrees of task complexity that are specifically tailored and sufficiently challenging relative to participants’ specific proficiency levels. Finally, the relatively small sample size, participants’ profiles (e.g., language proficiency, gender), and the local context limit the generalizability of the findings. Future research should examine the interplay among task complexity, task-specific emotions, and writing performance across more diverse educational contexts and different learner populations.

## Figures and Tables

**Table 1:** Descriptive Statistics for Simple Task and Complex Task.

Variable	Simple Task (N = 61)			Complex Task (N = 61)		
	M (SD)	Skewness (SE)	Kurtosis (SE)	M (SD)	Skewness (SE)	Kurtosis (SE)
Enjoyment	6.68 (1.32)	−0.08 (0.31)	−0.89 (0.60)	6.64 (1.13)	−0.17 (0.31)	0.84 (0.60)
Anxiety	3.98 (1.93)	−0.09 (0.31)	−1.02 (0.60)	4.03 (1.99)	0.26 (0.31)	−0.48 (0.60)
Boredom	2.78 (1.38)	0.52 (0.31)	−0.29 (0.60)	2.73 (1.29)	0.59 (0.31)	−0.65 (0.60)
Writing Performance	13.47 (1.89)	0.34 (0.31)	−0.38 (0.60)	13.14 (1.51)	0.62 (0.31)	1.18 (0.60)
Content	3.49 (0.61)	0.26 (0.31)	−0.19 (0.60)	3.30 (0.67)	0.24 (0.31)	0.33 (0.60)
Organization	3.79 (0.77)	−0.04 (0.31)	−0.66 (0.60)	3.64 (0.61)	−0.39 (0.31)	0.29 (0.60)
Language	3.10 (0.51)	0.54 (0.31)	0.78 (0.60)	3.11 (0.28)	2.55 (0.31)	5.39 (0.60)
Communicative Achievement	3.09 (0.52)	−0.01 (0.31)	0.34 (0.60)	3.09 (0.31)	2.05 (0.31)	4.37 (0.60)

**Table 2:** The Effects of Task Complexity on Learners’ L2 Writing Performance and Task-specific Emotions.

Variable	*t* (*df*)	*p* (Two-Tailed)	Mean Diff [95% CI]	*d*
Enjoyment	0.164 (60)	0.87	0.04 [−0.44, 0.52]	0.021
Anxiety	−0.133 (60)	0.894	−0.05 [−0.79, 0.69]	−0.017
Boredom	0.206 (60)	0.837	0.06 [−0.50, 0.61]	0.026
Writing Performance	1.384 (60)	0.172	0.33 [−0.15, 0.80]	0.177
Content	2.014 (60)	0.049 *	0.19 [0.00, 0.38]	0.258
Organization	1.321 (60)	0.192	0.15 [−0.08, 0.37]	0.169
Language	−0.127 (60)	0.899	−0.01 [−0.14, 0.12]	−0.016
Communicative Achievement	0.000 (60)	1	0.00 [−0.14, 0.14]	0

Note: * *p* < 0.05.

**Table 3:** Pearson Correlations Between Task-Specific Emotions and Overall Writing Performance Across Tasks.

Variable	1	2	3	4
Simple Task				
1. Writing Performance	1			
2. Enjoyment	0.083	1		
3. Anxiety	0.16	−0.260 *	1	
4. Boredom	−0.069	−0.530 **	0.164	1
Complex Task				
1. Writing Performance	1			
2. Enjoyment	0.036	1		
3. Anxiety	−0.284 *	−0.303 *	1	
4. Boredom	0.079	−0.617 **	0.031	1

Note: * *p* < 0.05, ** *p* < 0.01.

**Table 4:** Predictive Effect of Task-Specific Anxiety on Writing Performance in the Complex Task.

Predictor	*B*	*SE*	*β*	*t*	*p*	Tolerance	VIF
(Constant)	14.004	0.424		33.055	<0.001		
Anxiety	−0.214	0.094	−0.284	−2.272	0.027 *	1.000	1.000

Note: * *p* < 0.05. A stepwise regression method was employed, and enjoyment and boredom were tested but excluded from the final model due to non-significant contributions.

## Data Availability

The data presented in this study are available upon request from the corresponding author. The data are not publicly available because they consist of raw data that have been processed and analyzed for the purposes of this study.

## Abbreviations

The following abbreviations are used in this manuscript:

L2	Second Language
SLA	Second Language Acquisition
CAF	Complexity, Accuracy, and Fluency
EFL	English as a Foreign Language
ICC	Inter-rater Correlation Coefficient
VIF	Variance Inflation Factor

## Appendix A

### Appendix A.1. Simple Task (Adpated from Xu et al., 2023)

Imagine you can choose two best roommates from the four candidates when the new semester starts. Four properties of candidates: hobbies, personality, sleeping patterns, and studying style are listed in the table as follows.

Read the descriptions carefully and make a considered choice of the best roommates. Write an argumentative essay. Please provide at least 3 reasons to make your decision clear and convincing.

You have 40 min on this task. Please write at least 200 words.

The best roommates you choose: _________ & __________

Property	Nannan	Beibei	Niuniu	Dongdong
Hobbies	Watching movies; Listening to music; Reading books	Listening to music; Blogging; Playing the guitar	Photography; Playing football; Traveling	Listening to music; Playing football; Playing computer games
Personality	Introvert; Good at listening to others; Not good at communicating	Outgoing; Positive and enthusiastic; Happy to help others; Sometimes, ignoring others’ feelings unconsciously	Like playing with others; Not good at listening to others	Outgoing; Humorous; Weak self-discipline
Sleeping pattern	Early to bed, early to rise	Late to bed, early to rise	Early to bed, late to rise	Late to bed, late to rise
Studying style	He/she likes to study in a quiet place on his/her personal.	He/she likes to read out when he/she is studying.	He/she likes to study with others.	He/she likes to study on his/her personal.

### Appendix A.2. Complex Task (Adapted from Xu et al., 2023)

Imagine you can choose four best roommates from the six candidates when the new semester starts (two students in each dormitory). Six properties of candidates: hobbies, personality, sleeping patterns, studying style, favourite subjects, and individual sanitary habits are listed in the table as follows.

Read the descriptions carefully and make a considered choice of the best roommates. Write an argumentative essay. Please provide at least 3 reasons to make your decision clear and convincing.

You have 40 min on this task. Please write at least 200 words.

The best roommates you choose: _________ & __________,___________ & __________.

Property	Nannan	Beibei	Niuniu	Dongdong	Kangkang	Xuxu
Hobbies	Watching movies; Listening to music; Reading books	Listening to music; Blogging; Playing the guitar	Photography; Playing football; Traveling	Listening to music; Playing football; Playing computer games	Playing the piano; Listen to music; Watching movies	Playing football; Playing computer games; Playing magic
Personality	Introvert; Good at listening to others; Not good at communicating	Outgoing; Positive and enthusiastic; Happy to help others; Sometimes, ignoring others’ feelings unconsciously	Like playing with others; Not good at listening to others	Outgoing; Humorous; Weak self-discipline	Shy; Like to play with others; Weak self-discipline	Like to communicate with others and also good at listening to others. Outgoing, but sometimes kind of noisy
Sleeping pattern	Early to bed, early to rise	Late to bed, early to rise	Early to bed, late to rise	Late to bed, late to rise	Early to bed, late to rise	Late to bed, early to rise
Studying style	He/she likes to study in a quiet place on his/her personal.	He/she likes to read out when he/she is studying.	He/she likes to study with others.	He/she likes to study on his/her personal.	He/she likes to study in a quiet place on his/her personal.	He/she likes to read out in a low voice when he/she is studying.
Favourite subjects	English, Chinese	Math, Music	Sports, Arts	Computers, Music	Music English	Math, Computer
Individual sanitary habits	Tidy, but not good at cleaning	Good at cleaning	Neutral	Good at cleaning	Not good at cleaning	Neutral

### Appendix A.3. Rating Criteria

Band	Content - Relevance - Sufficient Information	Communicative Achievement - Style - Straightforward idea(concrete) - Complex idea (abstract) - Readers’ attention	Organization - Structure - Cohesive devices - Linking words	Language - Lexis: everyday words; less common words - Sentence: simple and complex - Accuracy
5	① All content is relevant: no irrelevant sentences. ② target reader is fully informed: at least 3 reasons are listed; the reasons are very convincing; all chosen roommates are involved; various dimensions of characteristics are considered.	① Uses the conventions of argumentative essay writing effectively to hold readers’ attention. ② Straightforward ideas and complex ideas are communicated appropriately.	Text is well organized and coherent, using a variety of cohesive devices, linking words, and organizational patterns (Cause-effect, clear structure) to generally good effect.	① Uses a range of vocabulary, including less common lexis, appropriately. ② Uses a range of simple and complex grammatical forms with control and flexibility. ③ Occasional errors (<3) may be present but do not impede communication.
4	Performance shares features of Band 3 and Band 5			
3	① Irrelevances may be present: 1–2 slightly irrelevant sentences. ② The target reader is, on the whole, informed: at least 3 reasons are listed; the reasons are rather convincing; all chosen roommates are involved	① Uses the conventions of argumentative essay writing to hold readers’ attention. ② Straightforward idea communicated appropriately.	Text is generally well organized and coherent, using a variety of linking words and cohesive devices, with a rather clear structure.	① Uses a range of everyday vocabulary appropriately, with occasional inappropriate use of less common lexis. ② Uses a range of simple and some complex grammatical forms with a good degree of control. ③ Errors do not impede communication.
2	Performance shares features of Band 1 and Band 3			
1	① Irrelevance and misinterpretation of the task, e.g., no roommates are chosen. ② Target reader is minimally informed: fewer than 3 reasons are listed, and/or the reasons are not convincing; the chosen roommate is not involved in the writing.	① Uses the conventions of an argumentative essay in generally appropriate ways to communicate straightforward ideas.	Text is connected and coherent, using basic linking words and a limited number of cohesive devices, but the structure is not clear.	① Uses everyday vocabulary generally appropriately, while occasionally overusing certain lexis. ② Uses simple grammatical forms with a good degree of control. ③ While errors are noticeable, meaning can still be determined.

## Appendix B

Instructions: Please recall the writing task you have just completed, read the following statements, and then give your ratings for each statement. Higher scores from 1 to 9 mean higher levels of agreement.

**Table A1:** *Task Specific Emotion Scales* (Adapted from C. Li, 2024; C. Li et al., 2024).

Scales	Items
Task-Specific Enjoyment	I enjoyed the task.
	I had a strong sense of accomplishment in the task.
	I was fully engaged in the task.
	I felt positive during the completion of the essay. The writing task was completed very smoothly.
Task-Specific Anxiety	I was anxious.
	I was terribly nervous.
	I worried about making mistakes.
	I was frustrated.
	I was trembling with fear.
Task-Specific Boredom	I felt bored.
	My mind was wandering.
	I did something irrelevant to the task.
	I didn’t know what to do.

## References

[B1-behavsci-16-00580] Abdi Tabari M., Khajavy G. H., Goetze J. (2024). Mapping the interactions between task sequencing, anxiety, and enjoyment in L2 writing development. Journal of Second Language Writing.

[B2-behavsci-16-00580] Abdi Tabari M., Lee H., Hanzawa K. (2025). Effects of massed and spaced task repetitions on L2 writing task performance and task emotions. Reading and Writing.

[B3-behavsci-16-00580] Adams R., Alwi N. A. N. M., Newton J. (2015). Task complexity effects on the complexity and accuracy of writing via text chat. Journal of Second Language Writing.

[B4-behavsci-16-00580] Aubrey S. (2017). Inter-cultural contact and flow in a task-based Japanese EFL classroom. Language Teaching Research.

[B5-behavsci-16-00580] Aubrey S. (2025). The relationship between task-specific anxiety, enjoyment, and use of planned content across discourse stages of second language learners’ spoken task performances. Learning and Individual Differences.

[B6-behavsci-16-00580] Aubrey S., Lambert C., Leeming P. (2022). The impact of first as opposed to second language pre-task planning on the content of problem-solving task performance. Language Teaching Research.

[B7-behavsci-16-00580] Cho M. (2018). Task complexity, modality, and working memory in L2 task performance. System.

[B8-behavsci-16-00580] Dewaele J.-M., Alfawzan M. (2018). Does the effect of enjoyment outweigh that of anxiety in foreign language performance?. Studies in Second Language Learning and Teaching.

[B9-behavsci-16-00580] Dewaele J.-M., MacIntyre P. D. (2014). The two faces of Janus? Anxiety and enjoyment in the foreign language classroom. Studies in Second Language Learning and Teaching.

[B10-behavsci-16-00580] Ellis R. (2003). Task-based language learning and teaching.

[B11-behavsci-16-00580] Flower L., Hayes J. (2004). A cognitive process theory of writing. College Composition and Communication.

[B12-behavsci-16-00580] Golparvar S. E., Rashidi F. (2021). The effect of task complexity on integrated writing performance: The case of multiple-text source-based writing. System.

[B13-behavsci-16-00580] Güvendir E., Uzun K. (2023). L2 writing anxiety, working memory, and task complexity in L2 written performance. Journal of Second Language Writing.

[B14-behavsci-16-00580] Hayes J., Indrisanio R., Squire J. (2000). A new framework for understanding cognition and affect in writing.

[B15-behavsci-16-00580] Heidari K. (2025). Cognitive fatigue induced by spaced and massed practice: Effects on idiom accuracy across L2 proficiency levels. International Review of Applied Linguistics in Language Teaching.

[B16-behavsci-16-00580] IBM Corp. (2022). IBM SPSS statistics *(Version 29) [Computer software]*.

[B17-behavsci-16-00580] Jung J. (2018). Effects of task complexity and working memory capacity on L2 reading comprehension. System.

[B18-behavsci-16-00580] Kline R. B. (2011). Principles and practice of structural equation modeling.

[B19-behavsci-16-00580] Lambert C., Zhang G. (2019). Engagement in the use of English and Chinese as foreign languages: The role of learner-generated content in instructional task design. The Modern Language Journal.

[B20-behavsci-16-00580] Lee J. (2020). Task closure and task complexity effects on L2 written performance. Journal of Second Language Writing.

[B21-behavsci-16-00580] Lewis M. D., Todd R., Xu X. (2010). The development of emotion regulation: A neuropsychological perspective. The handbook of life-span development.

[B22-behavsci-16-00580] Li C. (2024). Task-specific emotions in L2 writing: A control-value theory approach from a positive psychology perspective. Studies in Second Language Learning and Teaching.

[B23-behavsci-16-00580] Li C., Dewaele J.-M. (2024). Understanding, measuring, and differentiating task enjoyment from foreign language enjoyment.

[B24-behavsci-16-00580] Li C., Wei L. (2023). Anxiety, enjoyment, and boredom in language learning amongst junior secondary students in rural China: How do they contribute to L2 achievement?. Studies in Second Language Acquisition.

[B25-behavsci-16-00580] Li C., Wei L., Lu X. (2024). Task complexity and L2 writing performance of young learners: Contributions of cognitive and affective factors. The Modern Language Journal.

[B26-behavsci-16-00580] Li J., Wang J. (2017). A meta-analysis of task complexity effects on L2 writing: A report from RevMan. Foreign Language World.

[B27-behavsci-16-00580] Lightbown P., Spada N. (2013). How languages are learned.

[B28-behavsci-16-00580] Liu C., Sun L., He Y., Wu N. (2023). The effects of task complexity and language aptitude on EFL learners’ writing performance. Assessing Writing.

[B29-behavsci-16-00580] McRae K. (2009). The developmental trajectory of emotion regulation. Frontiers in Human Neuroscience.

[B30-behavsci-16-00580] Nakamura S., Phung L., Reinders H. (2021). The effect of learner choice on L2 task engagement. Studies in Second Language Acquisition.

[B31-behavsci-16-00580] Papi M., Manchón R. M., Polio C. (2022). The role of motivational and affective factors in L2 writing performance and written corrective feedback processing and use. The Routledge handbook of second language acquisition and writing.

[B32-behavsci-16-00580] Pawlak M., Kruk M., Elahi Shirvan M., Taherian T., Karimpour S. (2025). Dynamic reciprocal associations of AI-assisted L2 writing task emotions in data-driven learning: A dynamic structural equation modeling. Computer Assisted Language Learning.

[B33-behavsci-16-00580] Pekrun R. (2006). The control-value theory of achievement emotions: Assumptions, corollaries, and implications for educational research and practice. Educational Psychology Review.

[B34-behavsci-16-00580] Pekrun R., Perry R. P. (2014). Control-value theory of achievement emotions. International handbook of emotions in education.

[B35-behavsci-16-00580] Rahimi M. (2019). Effects of increasing the degree of reasoning and the number of elements on L2 argumentative writing. Language Teaching Research.

[B36-behavsci-16-00580] Rahimi M., Zhang L. J. (2018). Effects of task complexity and planning conditions on L2 argumentative writing production. Discourse Processes.

[B37-behavsci-16-00580] Rahimi M., Zhang L. J. (2019). Writing task complexity, students’ motivational beliefs, anxiety and their writing production in English as a second language. Reading and Writing.

[B38-behavsci-16-00580] R Core Team (2025). R: A language and environment for statistical computing *(Version 4.5.2) [Computer software]*.

[B39-behavsci-16-00580] Révész A., Kourtali N.-E., Mazgutova D. (2017). Effects of task complexity on L2 writing behaviors and linguistic complexity. Language Learning.

[B40-behavsci-16-00580] Richards J. C., Schmidt R. W. (2013). Longman dictionary of language teaching and applied linguistics.

[B41-behavsci-16-00580] Robinson P., Robinson P. (2001a). Task complexity, cognitive resources, and syllabus design: A triadic framework for examining task influences on SLA. Cognition and second language instruction.

[B42-behavsci-16-00580] Robinson P. (2001b). Task complexity, task difficulty, and task production: Exploring interactions in a componential framework. Applied Linguistics.

[B43-behavsci-16-00580] Robinson P., Pütz M., Sicola L. (2010). 13. Situating and distributing cognition across task demands: The SSARC model of pedagogic task sequencing. Converging evidence in language and communication research.

[B44-behavsci-16-00580] Shehadeh A., Coombe C., Davidson P., O’Sullivan B., Stoynoff S. (2012). Task-based language assessment: Components, development, and implementation. The Cambridge guide to second language assessment.

[B45-behavsci-16-00580] Shehadeh A., Liontas J. I. (2018). Task-based language assessment. The TESOL encyclopedia of English language teaching.

[B46-behavsci-16-00580] Shen Y., Zhang L. J., Sun Q. (2025). The effects of task complexity on L2 written production: A meta-analysis. Modern Foreign Languages.

[B47-behavsci-16-00580] Skehan P. (1998). A cognitive approach to language learning.

[B48-behavsci-16-00580] Tabari M. A., Lu X., Wang Y. (2023). The effects of task complexity on lexical complexity in L2 writing: An exploratory study. System.

[B49-behavsci-16-00580] Teng M. F., Zhan Y. (2023). Assessing self-regulated writing strategies, self-efficacy, task complexity, and performance in English academic writing. Assessing Writing.

[B50-behavsci-16-00580] Ten Hove D., Jorgensen T. D., van der Ark L. A. (2024). Updated guidelines on selecting an intraclass correlation coefficient for interrater reliability, with applications to incomplete observational designs. Psychological Methods.

[B51-behavsci-16-00580] Wilby J. (2020). Motivation, self-regulation, and writing achievement on a university foundation programme: A programme evaluation study. Language Teaching Research.

[B52-behavsci-16-00580] Wu L., Halim H. B. A. (2024). Task complexity and foreign language writing emotions as predictors of EFL writing performance. Frontiers in Education.

[B53-behavsci-16-00580] Xing J., Zhao H., Luo S., Zhang L. J. (2024). Task complexity, working memory, task emotions, and oral performance of university students. Modern Foreign Languages.

[B54-behavsci-16-00580] Xu T. S., Zhang L. J., Gaffney J. S. (2023). A multidimensional approach to assessing the effects of task complexity on L2 students’ argumentative writing. Assessing Writing.

[B55-behavsci-16-00580] Yang Y., Peng W., Chen G. (2025). Effects of achievement emotions on L2 writing performance in argumentative tasks with different complexity levels. Language Awareness.

